# Dupilumab improves pruritus and skin lesions in patients with prurigo nodularis: Pooled results from 2 phase 3 trials (LIBERTY-PN PRIME and PRIME2)

**DOI:** 10.1016/j.jdin.2024.03.025

**Published:** 2024-04-10

**Authors:** Gil Yosipovitch, Brian S. Kim, Shawn G. Kwatra, Nicholas K. Mollanazar, Sonja Ständer, Takahiro Satoh, Pedro Mendes-Bastos, Tsen-Fang Tsai, Elizabeth Laws, Michael C. Nivens, Jennifer Maloney, Genming Shi, Ashish Bansal, Ariane Dubost-Brama

**Affiliations:** a Dr Phillip Frost Department of Dermatology and Cutaneous Surgery and Miami Itch Center, University of Miami, Miami, Florida; b Department of Dermatology, Icahn School of Medicine at Mount Sinai, New York, New York; c Department of Dermatology, Johns Hopkins University School of Medicine, Baltimore, Maryland; d Department of Dermatology, University of Pennsylvania, Philadelphia, Pennsylvania; e Department of Dermatology and Center for Chronic Pruritus, University Hospital Münster, Mϋnster, Germany; f Department of Dermatology, National Defense Medical College, Tokorozawa, Japan; g Dermatology Center, Hospital CUF Descobertas, Lisbon, Portugal; h Department of Dermatology, National Taiwan University Hospital, Taipei City, Taiwan; i Sanofi, Bridgewater, New Jersey; j Regeneron Pharmaceuticals Inc, Tarrytown, New York; k Sanofi, Gentilly, France

**Keywords:** Dermatology Life Quality Index (DLQI), dupilumab, Investigator’s Global Assessment for Prurigo Nodularis Stage (IGA PN-S), prurigo nodularis, Worst Itch Numerical Rating Scale (WI-NRS)

## Abstract

**Background:**

Phase 3 PRIME/PRIME2 trials independently demonstrated efficacy and an acceptable safety profile of dupilumab adults with moderate-to-severe prurigo nodularis.

**Objective:**

To obtain a more precise estimate of onset and magnitude of treatment effect using PRIME/PRIME2 pooled data.

**Methods:**

In PRIME/PRIME2, patients were randomized to dupilumab or placebo for 24 weeks. Pooled analysis assessed proportion of patients achieving clinically meaningful improvement in itch, clear/almost-clear skin, or both; at weeks 12 and 24; overall and by demographic subgroups and changes from baseline to week 24 in symptoms, signs, and quality of life.

**Results:**

Patients receiving dupilumab (*n* = 153) vs placebo (*n* = 158) experienced significant improvements in all tested endpoints. At week 24, 90 (58.8%) dupilumab-treated vs 30 (19.0%) placebo-treated patients achieved clinically meaningful improvement in itch, 71 (46.4%) vs 27 (17.1%) clear/almost clear skin, and 54 (35.3%) vs 14 (8.9%) achieved both (*P* < .0001 for all). Treatment benefits were independent of baseline demographics. Safety to week 36 was generally consistent with the known dupilumab safety profile.

**Limitations:**

On-treatment data limited to 24 weeks.

**Conclusions:**

Pooled analysis confirmed improvements reported in individual trials and revealed earlier effect onset in itch and skin pain. Dupilumab treatment showed benefits across demographics.

**Capsule Summary**

• Similarly designed PRIME and PRIME2 randomized, placebo-controlled trials supported approval of dupilumab as the first systemic therapy in prurigo nodularis.
• The present pooled analysis of PRIME and PRIME2 data confirmed, with increased power, efficacy, onset of treatment effect, and acceptable safety profile of dupilumab in patients with moderate-to-severe prurigo nodularis.

## Introduction

Prurigo nodularis (PN) is a chronic inflammatory skin disease with severely pruritic skin nodules that substantially affects quality of life (QoL) and is associated with a high disease burden.^1^, ^2^, ^3^, ^4^, ^5^, ^6^

Topical therapies and systemic immunosuppressants or neuromodulators are used off-label, but often fail to control PN.^7^^,^^8^ The US Food and Drug Administration recently approved dupilumab as the first systemic PN therapy.^9^ Dupilumab is a fully human VelocImmune-derived monoclonal antibody that blocks the shared receptor component (interleukin 4 receptor alpha [IL-4Rα]) for interleukin (IL)-4 and IL-13,^10^^,^^11^ thus inhibiting signaling of these central drivers of type 2 inflammation.

LIBERTY-PN PRIME and PRIME2 were 2 similarly designed phase 3 trials of dupilumab in adults with moderate-to-severe PN.^12^ In each trial, dupilumab demonstrated significant improvements compared with placebo on prespecified endpoints, including itch, skin lesions, QoL, skin pain, and anxiety/depression, with safety generally consistent with its known safety profile.^12^ In the current study, to further explore the robustness and onset of dupilumab action in PN, we assessed changes from baseline to week 24 in skin symptoms, signs, QoL, anxiety/depression, and sleep, using pooled data from PRIME/PRIME2 trials. Additionally, we used the increased power of the pooled analysis to assess treatment effect by baseline demographic subgroups, and we report safety results up to week 36. Plain Language Summary available via Mendeley at https://data.mendeley.com/datasets/75xwz2849j/1.

## Methods

Details of individual PRIME/PRIME2 (NCT04183335/NCT04202679) trials have been published previously.^12^ PRIME and PRIME2 were randomized, placebo-controlled, phase 3 trials, conducted independently in 16 countries in North and South America, Europe, and Asia. Each trial included a 2 to 4-week screening period, a 24-week intervention period, and a 12-week posttreatment follow-up period (Supplementary Fig 1, available via Mendeley at https://data.mendeley.com/datasets/75xwz2849j/1). The studies were conducted in accordance with the Declaration of Helsinki, the International Conference on Harmonisation Good Clinical Practice guideline, and applicable regulatory requirements. All patients provided written informed consent before participating.

### Study design

This was a pooled analysis by treatment group of data from PRIME/PRIME2.

### Patients

Patients were adults (18-80 years) with moderate-to-severe PN inadequately controlled with topical therapies or for whom those therapies were inadvisable.

Key inclusion criteria were: PN diagnosed for ≥3 months prior to screening; average Worst Itch Numerical Rating Scale (WI-NRS) score ≥7 in the 7 days prior to day 1 (assessed daily); ≥20 pruriginous nodules at screening and day 1 (Investigator’s Global Assessment for PN-Stage [IGA PN-S] of 3 or 4), with involvement of at least 2 body areas; history of failure of a 2-week course of medium-to-superpotent topical corticosteroids (TCS), or TCS were not advisable. Detailed inclusion and exclusion criteria have been reported previously.^12^

### Treatment and procedures

Patients were randomized 1:1 to receive subcutaneous dupilumab 300 mg every other week (600 mg loading dose on day 1) or placebo for 24 weeks. Use of high-potency/superpotency TCS, systemic immunosuppressive and immunomodulating drugs, intralesional corticosteroid injections, and cryotherapy, phototherapy, opioid-receptor antagonists, gabapentin, pregabalin, and thalidomide were prohibited from 4 weeks prior to screening to the end of the trial. Patients on a stable regimen of low-to-medium potency TCS and topical calcineurin inhibitors prior to screening were allowed to continue their use throughout the trial. Patients on stable doses of antidepressants for 3 months prior to enrollment were eligible if they kept medication unchanged throughout the trial.

Atopy was defined as having a medical history of or ongoing atopic dermatitis (AD), allergic rhinitis/rhinoconjunctivitis, asthma, or food allergy. Atopic and nonatopic PN populations were capped at 60% of the trial population. Patients with ongoing mild AD were eligible; their enrollment was capped to 10% of the atopic population.

### Instruments used to assess efficacy

WI-NRS (0, “no itch” to 10, “worst imaginable itch”) is validated in PN, and a reduction of 4 points is considered clinically meaningful in patients with baseline WI-NRS ≥7.^13^, ^14^, ^15^ Patients reported a daily score; the average of the previous 7 days was used as the trial weekly WI-NRS score.

IGA PN-S, also validated in PN,^16^ is the clinician-assessed stage based on approximate number of palpable pruriginous nodules on a scale of 0-4 (0 [clear] = 0 nodules; 1 [almost clear] = 1-5; 2 [mild] = 6-19; 3 [moderate] = 20-99; 4 [severe] ≥100).

### Study endpoints

Efficacy endpoints addressing pruritus and skin lesions included proportion of patients with ≥4-point reduction from baseline in WI-NRS, or achieving IGA PN-S score 0 or 1, or both, at week 12 and week 24, and percent change in WI-NRS from baseline to week 24. Other efficacy endpoints included changes from baseline to week 24 in the Dermatology Life Quality Index (DLQI), Skin Pain-NRS, total Hospital Anxiety and Depression Scale, and Sleep Quality-NRS, as well as proportion of patients who used rescue medication through week 24.

Subgroup analyses included proportion of patients with ≥4-point reduction in WI-NRS, or IGA PN-S score 0/1, or both, at week 24, by age, sex, region, race, and baseline body mass index (BMI).

Safety was assessed up to week 36, including the treatment and follow-up periods.

### Statistical analysis

Efficacy analyses were performed on the full analysis set, including all randomized patients. For categorical endpoints, *P*-values and the response rate difference vs placebo (95% CI) were calculated by Cochran-Mantel-Haenszel test performed on the association between the responder status and intervention group, adjusted by randomization stratification factors (documented history of atopy [atopic/nonatopic], stable use of TCS or topical calcineurin inhibitors [yes/no], region, baseline antidepressant use [yes/no], and study indicator [PRIME/PRIME2]). Patients who received prohibited medications/procedures, rescue medication, or with missing data at the analysis timepoint were considered nonresponders. For continuous endpoints, data were analyzed by fitting an analysis of covariance model with the corresponding baseline value, intervention group, and randomization stratification factors as covariates. Data collected after study discontinuation were included. Data collected after prohibited medications/procedures, use of rescue medication, or study discontinuation due to lack of efficacy were set to missing and imputed by worst observation carried forward. Other missing data were imputed by multiple imputation. For subgroup analyses, odds ratios (OR) and 95% CI were derived from the Mantel-Haenszel estimator. ORs or 95% CIs for some specific subgroup categories could not be calculated due to either empty cell(s) or high imbalance within 1 or more cells of the 2 × 2 table (treatment vs improvement status) at each level of the stratification factors used in the analysis model. All reported *P* values are nominal.

All analyses were conducted using SAS software version 9.4.

## Results

### Patients

The pooled analysis included 311 randomized patients (placebo: 158; dupilumab: 153) (Supplementary Fig 2, available via Mendeley at https://data.mendeley.com/datasets/75xwz2849j/1). Baseline demographics and disease characteristics were balanced across treatment groups (Table I).

**Table I:** Demographic and clinical characteristics of the patient population at baseline

	Placebo *N* = 158	Dupilumab 300 mg q2w *N* = 153	Overall *N* = 311
Age (years), mean (SD)	48.8 (15.6)	50.1 (16.6)	49.5 (16.1)
Age group, *n* (%)			
<65 y	134 (84.8)	115 (75.2)	249 (80.1)
≥65 y	24 (15.2)	38 (24.8)	62 (19.9)
Weight (kg), mean (SD)	73.3 (18.5)	74.5 (17.3)	73.9 (17.9)
Female sex, *n* (%)	99 (62.7)	104 (68.0)	203 (65.3)
Race, *n* (%)			
White	93 (58.9)	83 (54.2)	176 (56.6)
Black or African American∗	8 (5.1)	11 (7.2)	19 (6.1)
Asian	52 (32.9)	54 (35.3)	106 (34.1)
Others or missing data†	5 (3.2)	5 (3.3)	10 (3.2)
Region‡, *n* (%)			
Asia	46 (29.1)	47 (30.7)	93 (29.9)
Eastern Europe	16 (10.1)	17 (11.1)	33 (10.6)
Latin America	30 (19.0)	25 (16.3)	55 (17.7)
Western countries	66 (41.8)	64 (41.8)	130 (41.8)
Duration of PN (y), mean (SD)	5.4 (6.6)	5.7 (7.2)	5.6 (7.0)
History of atopy§, *n* (%)	68 (43.0)	67 (43.8)	135 (43.4)
Ongoing mild AD‖	7 (4.4)	6 (3.9)	13 (4.2)
Stable use of TCS/TCI¶, *n* (%)	91 (57.6)	91 (59.5)	182 (58.5)
Prior topical medications for PN, *n* (%)	158 (100)	152¶¶ (99.3)	310 (99.7)
Prior systemic medications for PN, *n* (%)	104 (65.8)	102 (66.7)	206 (66.2)
Antihistamines	84 (53.2)	81 (52.9)	165 (53.1)
Corticosteroids	28 (17.7)	26 (17.0)	54 (17.4)
Nonsteroidal immunosuppressants	28 (17.7)	36 (23.5)	64 (20.6)
Antidepressants	15 (9.5)	11 (7.2)	26 (8.4)
WI-NRS# [0-10], mean (SD)	8.4 (1.1)	8.6 (0.9)	8.5 (1.0)
IGA PN-S∗∗ [0-4], *n* (%)			
3	102 (65.4)	103 (67.3)	205 (66.3)
4	54 (34.6)	50 (32.7)	104 (33.7)
DLQI†† [0-30], mean (SD)	17.0 (7.2)	18.0 (6.7)	17.5 (7.0)
Skin pain NRS‡‡ [0-10], mean (SD)	7.2 (2.4)	7.2 (2.5)	7.2 (2.4)
Sleep Quality NRS§§ [0-10], mean (SD)	4.2 (2.4)	4.4 (2.4)	4.3 (2.4)
Total HADS‖‖ [0-42], mean (SD)	15.1 (8.2)	15.4 (7.9)	15.2 (8.1)

*AD*, Atopic dermatitis; *DLQI*, Dermatology Life Quality Index; *HADS*, Hospital Anxiety and Depression Scale; *IGA PN-S*, Investigator’s Global Assessment for PN-Stage; *NRS*, Numerical Rating Scale; *PN*, prurigo nodularis; *q2w*, every 2 weeks; *TCI*, topical calcineurin inhibitors; *TCS*, topical corticosteroids; *WI-NRS*, Worst Itch Numerical Rating Scale.

∗ 36.4% (16/44) of US patients were Black or African American.

† Including American Indian or Alaska Native, Native Hawaiian or Pacific Islander, unknown.

‡ Asia: China, Japan, South Korea, Taiwan; Eastern Europe: Hungary, Russia; Latin America: Argentina, Chile, Mexico; Western countries: USA, Canada, France, Italy, Portugal, Spain, and UK.

§ Defined as having a medical history of atopic dermatitis, allergic rhinitis/rhinoconjunctivitis, asthma, food allergy. Population with history of atopy was capped at 60% of the trial population.

‖ Mild ongoing atopic dermatitis population was capped at 10% of the atopic population.

¶ Defined as maintaining the same medicine (low-to-medium potency TCS or TCI) and the same frequency of treatment (once or twice daily) used from 2 weeks prior to screening.

# Worst itch in the last 24 hours, from 0 = no itch, to 10 = worst imaginable itch.

∗∗ 0 (clear) = no palpable pruriginous nodules, 1 (almost clear) = approximately 1-5 palpable nodules, 2 (mild) = approximately 6-19 palpable nodules, 3 (moderate) = approximately 20-99 palpable nodules, 4 (severe) abundant, ≥100 palpable nodules.

†† Effect of PN on quality of life: 0-1 = no effect, 2-5 = small, 6-10 = moderate, 11-20 = very large, and 21-30 = extremely large effect.

‡‡ Worst skin pain in the last 24 hours, from 0 = no pain, to 10 = worst imaginable pain.

§§ Best description of sleep quality in the last 24 hours, from 0 = best possible sleep, to 10 = worst possible sleep.

‖‖ Scale for both anxiety and depression is 0-21; ≤7 = normal, 8-10 = possible case, ≥11 = probably case.

¶¶ One patient who was initially considered as not having used TCS prior to enrolment was confirmed as having used TCS post-database lock, bringing the overall proportion of patients with prior TCS use to 100%.

Prior to enrollment, all patients had used topical medications for PN, and 66.2% had used systemic medications. Despite prior therapies, at baseline, all patients had moderate or severe disease as defined by pruriginous nodule count, severe or very severe itch, skin pain, and impaired sleep. The overall mean (SD) WI-NRS score was 8.5 (1.0). Among enrolled patients, 66.3% had 20-99 nodules (IGA PN-S = 3), and 33.7% had ≥100 nodules (IGA PN-S = 4). Mean (SD) Skin Pain-NRS was 7.2 (2.4), and Sleep Quality-NRS was 4.3 (2.4). Mean (SD) DLQI score was 17.5 (7.0), indicating a very large effect of disease on QoL.

Among enrolled patients, 83.6% had comorbidities and/or surgery history, predominantly vascular (27.0%), metabolism and nutrition (25.1%), and gastrointestinal disorders (19.6%), and 10.9% had a medical history of neoplasms (Supplementary Table I, available via Mendeley at https://data.mendeley.com/datasets/75xwz2849j/1). The most common conditions associated with PN were hypertension (22.5%), type 2 diabetes mellitus (9.3%), and hypothyroidism (8.7%). Overall, 43.4% had history of atopy, including 4.2% with ongoing mild AD. Fifty patients (16.1%) had psychiatric comorbidities, including 22 (7.1%) with depressive disorders (Supplementary Table II, available via Mendeley at https://data.mendeley.com/datasets/75xwz2849j/1).

### Efficacy on pruritus and skin lesions

**Fig 1:**
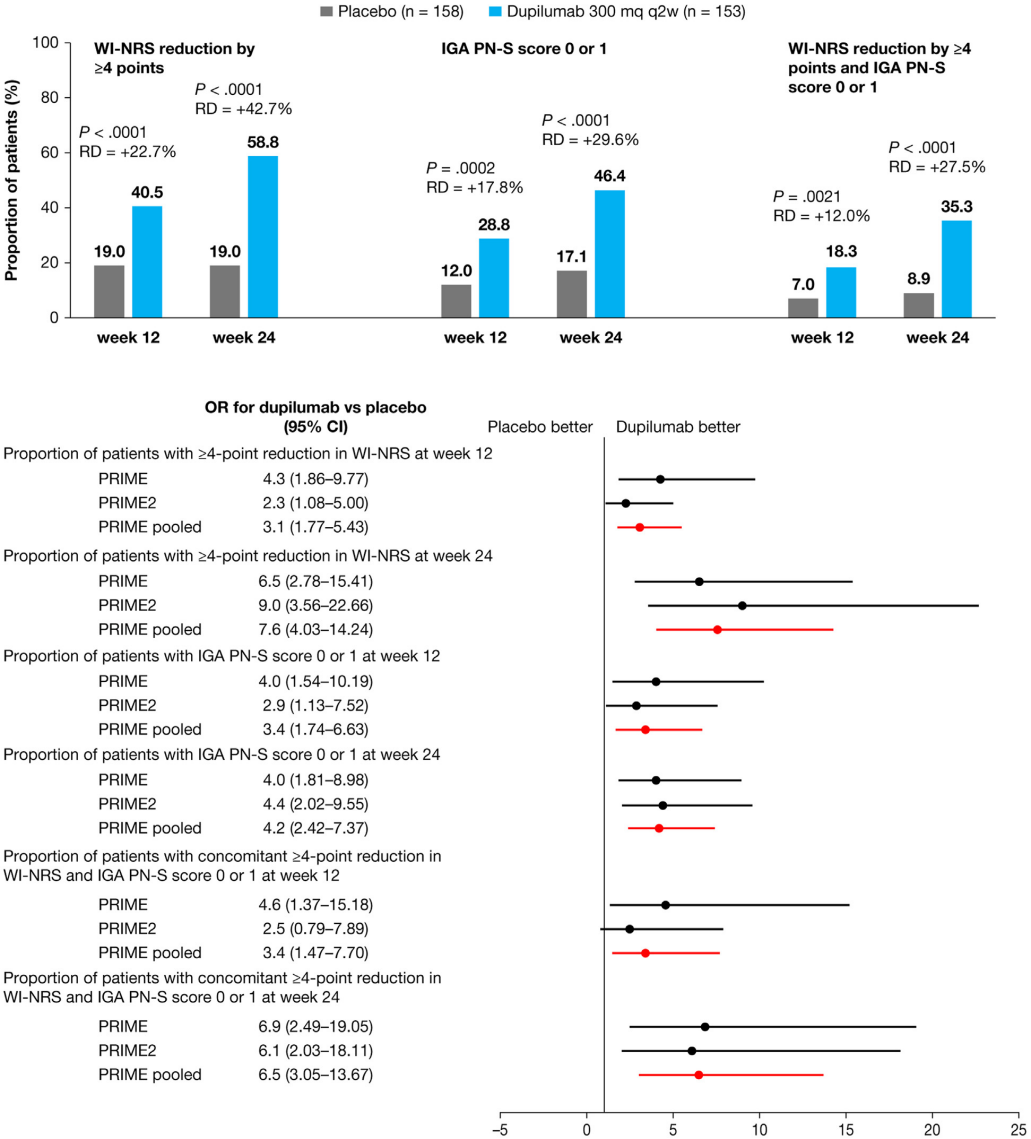
Prurigo nodularis. Proportion of patients with ≥4-point reduction from baseline in WI-NRS, IGA PN-S score 0 or 1, or both, at week 12 and week 24 in the pooled analysis (*top*), and comparison with individual trials (*bottom*). Cochran-Mantel-Haenszel test was performed on the association between the responder status and intervention group, adjusted by documented history of atopy (atopic or nonatopic), stable use of TCS/TCI (yes or no), region, baseline antidepressant use (yes or no), and study indicator (PRIME and PRIME2). ORs were derived from the Mantel-Haenszel estimator. *CI*, Confidence interval; *IGA PN-S*, Investigator’s Global Assessment for PN-Stage (range 0-4); *OR*, odds ratio; *q2w*, every 2 weeks; *RD*, raw difference; *TCI*, topical calcineurin inhibitors; *TCS*, topical corticosteroids; *WI-NRS*, Worst Itch Numerical Rating Scale (range 0-10).

**Fig 2:**
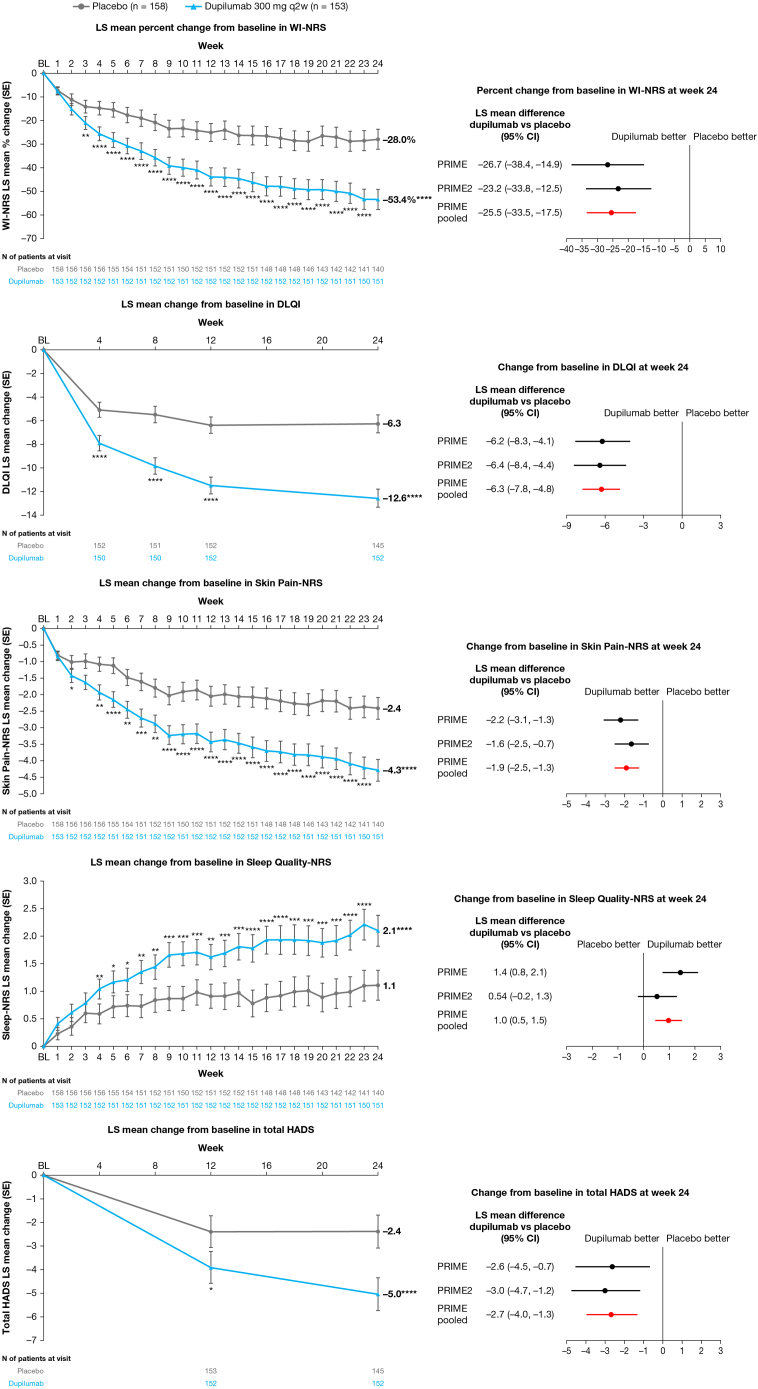
Prurigo nodularis. Patient-reported outcomes over time in the pooled analysis (*left*) and comparison of results at week 24 with individual trials (*right*). ∗*P* < .05; ∗∗*P* < .01; ∗∗∗*P* < .001; ∗∗∗∗*P* < .0001. Each of the imputed complete data were analyzed by fitting an analysis of covariance model with the corresponding baseline value, intervention group, documented history of atopy (atopic or nonatopic), stable use of TCS/TCI (yes or no), region, baseline antidepressant use (yes or no), and the study indicator (PRIME or PRIME2) as covariates. *DLQI*, Dermatology Life Quality Index; *HADS*, Hospital Anxiety and Depression Scale; *LS*, least-squares; *NRS*, Numerical Rating Scale; *TCI*, topical calcineurin inhibitors; *TCS*, topical corticosteroids; *WI-NRS*, Worst-Itch Numerical Rating Scale.

Clinically meaningful ≥4-point reduction in WI-NRS was achieved by 62 patients (40.5%) at week 12 and by 90 (58.8%) at week 24 in the dupilumab group vs 30 patients (19.0%) at both timepoints in the placebo group (*P* < .0001 for both) (Fig 1). IGA PN-S score 0/1 was achieved by 44 patients (28.8%) in the dupilumab group vs 19 (12.0%) in the placebo group at week 12 (*P* = .0002), and, respectively, by 71 (46.4%) vs 27 (17.1%) at week 24 (*P* < .0001) (Fig 1). Concomitant reduction in WI-NRS by ≥4 points and IGA PN-S score 0/1 (composite endpoint) was achieved by 28 (18.3%) dupilumab-treated patients vs 11 (7.0%) in the placebo group at week 12 (*P* = .0021), and, respectively, by 54 (35.3%) vs 14 (8.9%) at week 24 (*P* < .0001) (Fig 1). Dupilumab treatment was superior to placebo in achieving clinically meaningful itch reduction, clear or almost clear skin, or both, at week 24, irrespective of age, sex, geographic region, race, or BMI (Supplementary Fig 3, available via Mendeley at https://data.mendeley.com/datasets/75xwz2849j/1). Proportion of patients achieving ≥4-point reduction in WI-NRS from baseline was significantly higher vs placebo from week 2 (*P* = .0069) (Supplementary Fig 4, available via Mendeley at https://data.mendeley.com/datasets/75xwz2849j/1), and percent change in WI-NRS from baseline from week 3 (*P* = .0076) to week 24 (Fig 2).

### Other patient-reported outcomes

Pooled analysis confirmed results seen in individual trials for DLQI, skin pain, and anxiety and depression, with increased precision of the effect size (Fig 2). Improvement in Sleep Quality-NRS had not reached statistical significance in PRIME2; consistent with PRIME, pooled analysis demonstrated significant improvement in sleep quality in dupilumab-treated patients compared with placebo (Fig 2). Mean changes from baseline were significant vs placebo starting at week 2 for Skin Pain-NRS, week 4 for DLQI and Sleep Quality-NRS, and week 12 for total Hospital Anxiety and Depression Scale, and improvements were sustained to week 24 (Fig 2). At week 24, all tested patient-reported outcomes had improved in the dupilumab group to mean scores corresponding to small-to-moderate impairment (Supplementary Fig 5, available via Mendeley at https://data.mendeley.com/datasets/75xwz2849j/1).

### Use of rescue medication

**Fig 3:**
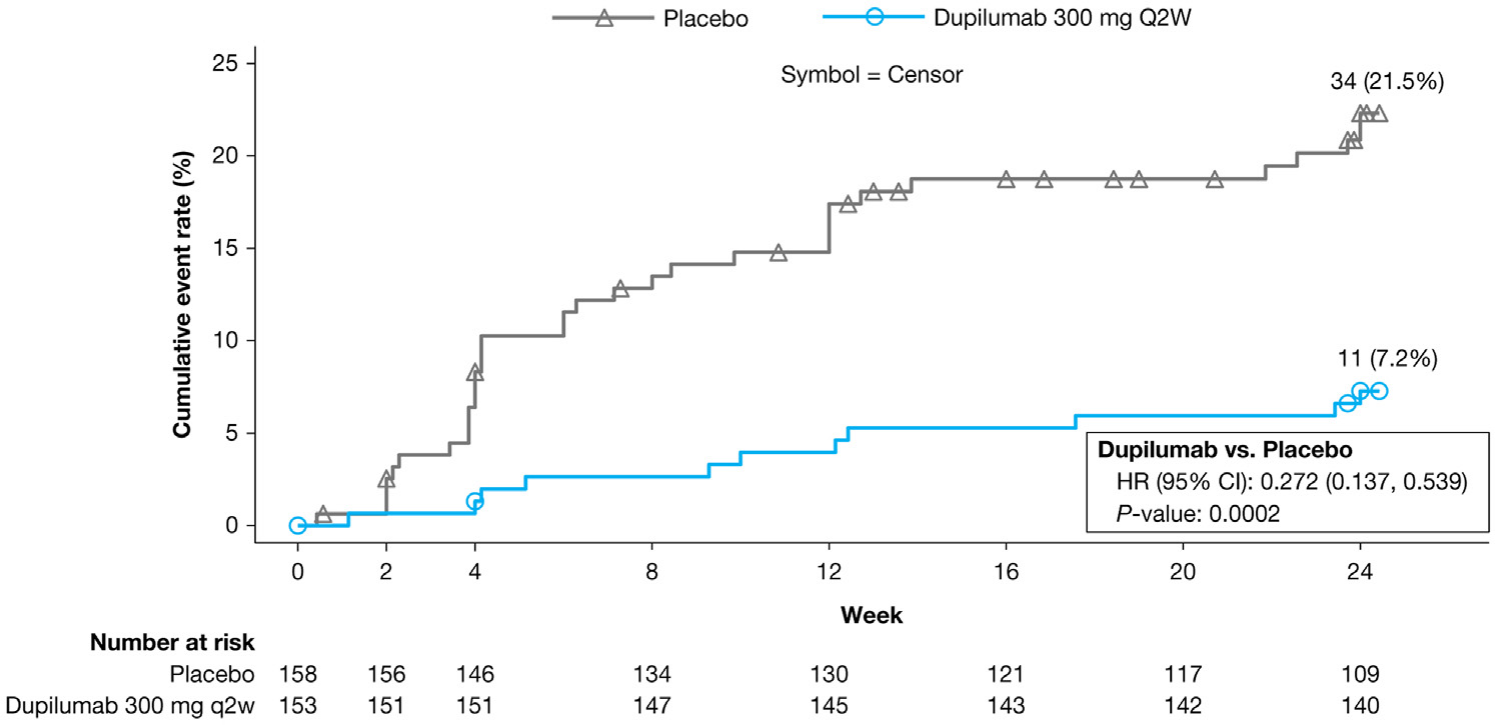
Prurigo nodularis. Kaplan-Meier curve of time to first use of high potency or superpotent topical corticosteroid rescue medication through week 24. *CI*, Confidence interval; *HR*, hazard ratio; *q2w*, every 2 weeks.

Fewer patients in the dupilumab group (11 [7.2%]) vs placebo (36 [22.8%]) needed rescue medication during the 24-week treatment period (Supplementary Table III, available via Mendeley at https://data.mendeley.com/datasets/75xwz2849j/1). The cumulative rate of symptom breakthrough requiring TCS rescue medication separated early for dupilumab vs placebo (week 2) and was significantly lower for the dupilumab group vs placebo over the remainder of the 24-week treatment period (Fig 3).

### Safety outcomes

Dupilumab was well tolerated, with safety generally consistent with its known safety profile. Treatment-emergent adverse events (TEAEs) were reported in 97 patients (63.8%) treated with dupilumab and 89 (56.7%) receiving placebo, and serious TEAEs were reported in 4.6% and 7.6%, respectively. The most common TEAEs were headache (dupilumab 5.3% vs placebo 5.7%) and neurodermatitis (2.6% vs 7.0%). No deaths occurred in either study, and TEAEs leading to treatment discontinuation were rare (none with dupilumab, and 4 [2.5%] with placebo) (Table II). Six patients (3.9%) receiving dupilumab vs 2 (1.3%) receiving placebo experienced events of conjunctivitis, which were not serious and did not lead to treatment discontinuation. Incidence of herpes viral infections was higher in the dupilumab group (3.3% vs 0), while skin infections were numerically greater with placebo (8.9%) than with dupilumab (4.6%) (Table II).

**Table II:** Safety overview for the 36-week treatment and follow-up periods

	Placebo *n* = 157∗ *n* (%)	Dupilumab 300 mg q2w *n* = 152† *n* (%)
Patients with any TEAE	89 (56.7)	97 (63.8)
Patients with any treatment-emergent SAE	12 (7.6)	7 (4.6)
Patients with any TEAE leading to death	0	0
Patients with any TEAE leading to permanent discontinuation of study drug‡	4 (2.5)	0
TEAEs reported in ≥5% of patients in any treatment group (MedDRA PT)§		
Headache	9 (5.7)	8 (5.3)
Neurodermatitis	11 (7.0)	4 (2.6)
Other AEs of interest		
Skin infections (excluding herpetic infections)‖	14 (8.9)	7 (4.6)
Conjunctivitis (narrow)¶	2 (1.3)	6 (3.9)
Herpes viral infections (HLT)#	0	5 (3.3)
Severe or serious infection∗∗	3 (1.9)	2 (1.3)
COVID-19 (PT)	5 (3.2)	1 (0.7)
Systemic hypersensitivity reactions (medically reviewed)††	2 (1.3)	1 (0.7)
Malignancy‡‡	2 (1.3)	1 (0.7)
Drug-related hepatic disorder	3 (1.9)	0
Keratitis	0	0

*AE*, Adverse event; *HLT*, MedDRA High Level Term; *IMP*, investigational medicinal product; *MedDRA*, Medical Dictionary for Regulatory Activities; *PT*, MedDRA Preferred Term; *SAE*, serious adverse event; *TEAE*, treatment-emergent adverse event.

∗ One patient included in the PRIME trial was randomized but not treated with IMP due to fear of being exposed to COVID-19.

† One patient included in the PRIME2 trial was not treated with IMP due to participant’s decision.

‡ One event each of Hodgkin’s disease, duodenal ulcer perforation, and neurodermatitis, considered IMP unrelated, and 1 event of urticaria, considered IMP related.

§ Accidental overdose, defined as twice the intended dose during an interval of 11 days, was reported as a TEAE in 7 (4.5%) placebo- and 9 (5.9%) dupilumab-treated patients; these events represented shorter interval between injection administration than the interval stated in the protocol and not an increased dose. None of the events was symptomatic, and none associated with medical consequences.

‖ Skin infections TEAE (excluding herpetic infections) were identified based on blinded medical review of all reported TEAEs identified as possible skin infections using CMQ30067 (Company MedDRA Query). This search included PTs under MedDRA High Level Group Term skin and subcutaneous tissue infections and infestations, all PTs under HLT skin structures and soft tissue infections, all PTs of “wound infection,” and PTs of chalazion, hordeolum, and skin papilloma.

¶ Conjunctivitis (narrow term) includes the PTs conjunctivitis, conjunctivitis bacterial, conjunctivitis viral, conjunctivitis adenoviral, conjunctivitis allergic, and atopic keratoconjunctivitis.

# Herpes viral infections (HLT) includes the PTs oral herpes, herpes zoster, ophthalmic herpes zoster, genital herpes simplex.

∗∗ In the placebo group, 1 event of severe COVID-19 and 1 event of cellulitis, considered IMP unrelated, and one event of sepsis, considered IMP related; in the dupilumab group, 1 event of moderate COVID-19 pneumonia and one event of pyelonephritis acute, considered IMP unrelated; none of the infections led to permanent discontinuation.

†† In the placebo group, 2 events considered IMP related, 1 nonserious event of moderate urticaria, not leading to study discontinuation, and 1 nonserious event of mild urticaria, which led to permanent discontinuation; in the dupilumab group, 1 nonserious event of mild dermatitis allergic, considered IMP unrelated and not leading to discontinuation.

‡‡ In the placebo group, 1 event of Hodgkin’s lymphoma, and 1 event of cutaneous T cell lymphoma; in the dupilumab group, 1 event of papillary thyroid carcinoma; all were considered IMP unrelated and led to permanent study drug discontinuation.

## Discussion

The present analysis confirms individual trial results, adds information on efficacy by demographic subgroups, and extends previously reported safety up to week 36. Dupilumab demonstrated efficacy vs placebo in all tested efficacy endpoints, including clinically meaningful reduction in itch, reduction in pruriginous nodule number to ≤5, reduction in skin pain and anxiety/depression, and improvement in sleep quality and QoL. Pooling data from the 2 PRIME studies allowed for greater precision in delineating the onset of action and revealed earlier differentiation from placebo in WI-NRS mean percent change from baseline (week 3, consistent with PRIME) than that seen in the PRIME2 trial (week 4), as well as in Skin Pain-NRS mean change from baseline (week 2, compared with week 3 in PRIME and week 4 in PRIME2). Additionally, consistent with results in PRIME, but in contrast to PRIME2, the pooled analysis showed nominally significant improvements in sleep quality from week 4 to week 24.^12^

Analyses by baseline demographic subgroups showed a consistent trend of dupilumab benefit compared with placebo in achieving ≥4-point reduction in WI-NRS, IGA PN-S 0 or 1, or both, at week 24, with comparable effect size in all demographic subgroups that included sufficient patients for meaningful testing. Racial differences have been demonstrated in PN, with more males affected in the Asian population and more females affected in White and African-American populations.^17^^,^^18^ African-American patients are at higher risk of developing PN and typically experience more severe itch, greater thickening and tissue fibrosis, worse disease control, and greater systemic inflammation compared with White patients.^19^, ^20^, ^21^ In our study, the magnitude of the treatment effect on itch improvement and skin nodule clearance was comparable between White and Asian patients. The percentage of responders vs placebo in the Black patient subgroup also showed dupilumab benefit (45.5% vs 12.5% for itch and 36.4% vs 12.5% for skin lesions); however, ORs could not be calculated due to the small sample size.

The safety profile was generally consistent with previous dupilumab trials.^22^, ^23^, ^24^, ^25^ There were no keratitis events. The incidence of conjunctivitis in dupilumab-treated patients was 3.9% (including 1 patient with comorbid mild AD). This incidence is lower than that reported in AD trials (9.3% in AD 16-week monotherapy trials with 300 mg dupilumab every 2 weeks; 13.6% in 52-week CHRONOS and 28% in 16-week CAFÉ AD trials of dupilumab with concomitant TCS),^26^ which is in line with previous studies showing higher incidence of ocular surface disorders in AD clinical trials compared with other dupilumab indications.^26^ Consistent with previous dupilumab trials for AD,^27^, ^28^, ^29^ in this analysis, fewer patients in the dupilumab group vs placebo developed non-herpetic skin infections (7 [4.6%] vs 14 [8.9%]). This may be explained by the rapid effect of dupilumab in breaking the itch/scratch cycle,^30^ its restorative effect on skin barrier integrity,^31^ and its targeted IL-4/IL-13 inhibition without broad immunosuppression.

The itch-scratch cycle in PN is believed to be driven by T helper/type 2 innate lymphoid/mast cells and IL-4 and Il-13 cytokines.^32^, ^33^, ^34^, ^35^ Signaling through IL-4Rα, which is expressed on various subsets of itch sensory neurons,^33^^,^^34^ was shown to sensitize neurons to other pruritogens released in inflamed skin.^33^^,^^35^ Therefore, IL-4Rα blockade with dupilumab is expected to alter the itch-scratch cycle in PN. Dupilumab may also impact the activity of IL-31, an important driver of pruritus, as IL-4 stimulation upregulates IL-31 receptor A expression and is the central driver of T helper 1 or 2 cell precursor polarization. As T helper 2 cells are the main source of IL-31, IL-4/IL-13 blockade with dupilumab could potentially prevent activation of sensory neurons by reducing IL-31 signaling.^33^

Limitations of this study include the relatively short treatment duration and the small representation of the Black/African-American population (6.1%). Given that all tested efficacy endpoints improved progressively over 24 weeks, longer treatment may lead to a sustained itch-free state, which in turn may curb emergence of nodules and allow skin remodeling and healing.

In conclusion, this pooled analysis confirmed findings from individual trials and, through its increased power, supported improvements in sleep quality, pointed to earlier onset of action in itch and skin pain, and showed clinical efficacy across age, sex, race, and baseline BMI. Safety was generally consistent with the known dupilumab safety profile.

## Data availability

Qualified researchers may request access to patient-level data and related study documents, including the clinical study report, study protocol with any amendments, blank case report form, statistical analysis plan, and dataset specifications. Patient-level data will be anonymized, and study documents will be redacted to protect the privacy of our trial participants.

## Conflicts of interest

Yosipovitch G: AbbVie, Arcutis Biotherapeutics, Bellus Health, Celldex, Eli Lilly, Escient Pharmaceuticals, Galderma, GSK, Kiniksa Pharmaceuticals, LEO Pharma, Novartis, Pfizer, Pierre Fabre, Regeneron Pharmaceuticals Inc, Sanofi, Trevi Therapeutics – advisory board member/consultant; Eli Lilly, Kiniksa Pharmaceuticals, LEO Pharma, Novartis, Pfizer, Regeneron Pharmaceuticals Inc, Sanofi – grants/research funding; Regeneron Pharmaceuticals Inc, Sanofi – investigator. Kim BS: Klirna Biotech – co-founder; 23andMe, Abrax Japan, AbbVie, Almirall, Amagma, Amgen, Arcutis Biotherapeutics, Arena Pharmaceuticals, Argenx, AstraZeneca, Bellus Health, Blueprint Medicines, Boehringer Ingelheim, Bristol Myers Squibb, Cara Therapeutics, Clexio Biosciences, CymaBay Therapeutics, Eli Lilly, Escient Pharmaceuticals, Evommune, Galderma, Genentech, Granular Therapeutics, GSK, Guidepoint Global, Incyte, Innovaderm Research, Janssen Pharmaceuticals, Kiniksa Pharmaceuticals, LEO Pharma, Medicxi, Micreos, Novartis, OM Pharma, Pfizer, RecensMedical, Regeneron Pharmaceuticals Inc, Sanofi, Septerna, Shaperon, Teva, Third Harmonic Bio, Third Rock Ventures, Trevi Therapeutics, Triveni, Vial, WebMD – consultant; Cara Therapeutics, Celgene, Kiniksa Pharmaceuticals, Menlo Therapeutics, Regeneron Pharmaceuticals Inc, Sanofi, Theravance Biopharma – advisory board member; Abrax Japan, Klirna Biotech, Locus Biosciences, Nuogen Pharma, RecensMedical – stockholder; patent holder for the use of JAK1 inhibitors for chronic pruritus; patent pending for the use of JAK inhibitors for interstitial cystitis; Nuogen Pharma – founder, chief scientific officer; Cara Therapeutics, LEO Pharma – research grants. Kwatra SG: AbbVie, Arcutis Biotherapeutics, Aslan Pharmaceuticals, Celldex Therapeutics, Galderma, Genzada Pharmaceuticals, Incyte, Johnson & Johnson, Kiniksa Pharmaceuticals, Novartis, Pfizer, Regeneron Pharmaceuticals Inc, Sanofi, Trevi Therapeutics – advisory board member/consultant; Galderma, Incyte, Pfizer, Sanofi – investigator. Mollanazar NK: AbbVie, Boehringer Ingelheim, Galderma, Janssen, LEO Pharma, Novartis, Regeneron Pharmaceuticals Inc, Sanofi, Trevi Therapeutics – advisory board member; Regeneron Pharmaceuticals Inc, Sanofi – investigator. Ständer S: Celldex Therapeutics, Clexio Biosciences, Dermasence, Galderma, GSK, Incyte, Kiniksa Pharmaceuticals, Menlo Therapeutics, Novartis, Sanofi, Trevi Therapeutics – investigator; AbbVie, Almirall, Beiersdorf, Bellus Health, BenevolentAI, Bionorica, Bristol Myers Squibb, Cara Therapeutics, Cello Health, Clexio Biosciences, DS Biopharma, Eli Lilly, Escient Pharmaceuticals, Galderma, Incyte, Integrity CE, Grünenthal, Kiniksa Pharmaceuticals, Klinge Pharma, Klirna Biotech, Menlo Therapeutics, Perrigo, Pfizer, Professor Paul Gerson Unna Academy, Sanofi, Siena Biopharmaceuticals, Touch IME, Trevi Therapeutics, Vanda Pharmaceuticals, Vifor Pharma, WebMD – consultant/advisory board; AbbVie, Almirall, Beiersdorf, Bristol Myers Squibb, Eli Lilly, FOMF, Galderma, LEO Pharma, L’Oréal, MEDahead, Menlo Therapeutics, Novartis, Omnicuris, Pfizer, Pierre Fabre, Professor Paul Gerson Unna Academy, Sanofi, UCB, Vifor Pharma – speaker. Satoh T: AbbVie, Asahi Kasei, Bristol Myers Squibb, Eli Lilly, Hisamitsu Pharmaceutical, Kracie, Kyorin Pharmaceutical, Maruho, Mitsubishi Tanabe Pharma, Otsuka Pharmaceutical, Pfizer, Sanofi, Torii Pharmaceutical – speaker; AbbVie, Daiichi Sankyo, Eli Lilly Japan K. K., Kaken Pharmaceutical, Kyowa Hakko Kirin, Nippon Zoki Pharmaceutical, Otsuka Pharmaceutical, Sato Pharmaceutical, Sun Pharma, Taiho Pharmaceutical, Torii Pharmaceutical – research grants; Nankodo – other fees; Sanofi – investigator. Mendes-Bastos P: AbbVie, Bayer, Cantabria Labs, CS Laboratórios, Eli Lilly, Evelo Biosciences, Janssen-Cilag, LEO Pharma, L’Oréal, Novartis, Organon, Pfizer, Pierre Fabre, Sanofi, Teva, Viatris – speaker/advisor/consultant; AbbVie, Janssen-Cilag, Novartis, Pfizer, Sanofi – Principal Investigator in clinical trials. Tsai TF: AbbVie, AnaptysBio, Boehringer Ingelheim, Bristol Myers Squibb, Celgene, Eli Lilly, Galderma, GSK, Janssen-Cilag, LEO Pharma, Merck Sharp & Dohme, Novartis, Pfizer, PharmaEssentia, Sanofi, Sun Pharma, UCB Pharma – investigator/consultant. Laws E, Shi G, Dubost-Brama A: Sanofi – employees, may hold stock and/or stock options in the company. Nivens MC, Maloney J, Bansal A: Regeneron Pharmaceuticals Inc – employees and shareholders.

## References

[1] Aggarwal P., Choi J., Sutaria N. (2021). Clinical characteristics and disease burden in prurigo nodularis. Clin Exp Dermatol.

[2] Elmariah S., Kim B., Berger T. (2021). Practical approaches for diagnosis and management of prurigo nodularis: United States expert panel consensus. J Am Acad Dermatol.

[3] Whang K.A., Le T.K., Khanna R. (2022). Health-related quality of life and economic burden of prurigo nodularis. J Am Acad Dermatol.

[4] Steinke S., Zeidler C., Riepe C. (2018). Humanistic burden of chronic pruritus in patients with inflammatory dermatoses: results of the European Academy of Dermatology and Venereology network on assessment of severity and burden of pruritus (PruNet) cross-sectional trial. J Am Acad Dermatol.

[5] Zeidler C., Pereira M.P., Dugas M. (2021). The burden in chronic prurigo: patients with chronic prurigo suffer more than patients with chronic pruritus on non-lesional skin: a comparative, retrospective, explorative statistical analysis of 4,484 patients in a real-world cohort. J Eur Acad Dermatol Venereol.

[6] Wongvibulsin S., Sutaria N., Williams K.A. (2021). A nationwide study of prurigo nodularis: disease burden and healthcare utilization in the United States. J Invest Dermatol.

[7] Williams K.A., Roh Y.S., Brown I. (2021). Pathophysiology, diagnosis, and pharmacological treatment of prurigo nodularis. Expert Rev Clin Pharmacol.

[8] Qureshi A.A., Abate L.E., Yosipovitch G. (2019). A systematic review of evidence-based treatments for prurigo nodularis. J Am Acad Dermatol.

[9] DUPIXENT® (dupilumab) (2022). Highlights of prescribing information. US Food and Drug Administration. https://www.accessdata.fda.gov/drugsatfda_docs/label/2020/761055s020lbl.pdf.

[10] Macdonald L.E., Karow M., Stevens S. (2014). Precise and in situ genetic humanization of 6 Mb of mouse immunoglobulin genes. Proc Natl Acad Sci U S A.

[11] Murphy A.J., Macdonald L.E., Stevens S. (2014). Mice with megabase humanization of their immunoglobulin genes generate antibodies as efficiently as normal mice. Proc Natl Acad Sci U S A.

[12] Yosipovitch G., Mollanazar N., Ständer S. (2023). Dupilumab in patients with prurigo nodularis: two randomized, double-blind, placebo-controlled phase 3 trials. Nat Med.

[13] Ständer S., Zeidler C., Pereira M. (2022). Worst itch numerical rating scale for prurigo nodularis: a psychometric evaluation. J Eur Acad Dermatol Venereol.

[14] Riepe C., Osada N., Reich A. (2019). Minimal clinically important difference in chronic pruritus appears to be dependent on baseline itch severity. Acta Derm Venereol.

[15] Bahloul D, Thomas RB, Rhoten S, et al. Validation of the Worst-Itch Numeric Rating Scale (WI-NRS) in prurigo nodularis (PN) based on clinical studies of dupilumab in adults with PN. Poster presented at AAD 2023. March 17-21, New Orleans, LA.

[16] Zeidler C., Pereira M.P., Augustin M. (2021). Investigator's global assessment of chronic prurigo: a new instrument for use in clinical trials. Acta Derm Venereol.

[17] Boozalis E., Tang O., Patel S. (2018). Ethnic differences and comorbidities of 909 prurigo nodularis patients. J Am Acad Dematol.

[18] Woo Y.-R., Wang S., Sohn K.-A. (2021). Epidemiology, comorbidities, and prescription patterns of Korean prurigo nodularis patients: a multi-institution study. J Clin Med.

[19] Kwatra S.G. (2022). Prurigo nodularis. JAMA Dermatol.

[20] Sutaria N., Alphonse M.P., Roh Y.S. (2022). Cutaneous transcriptomic identifies fibroproliferative and neurovascular gene dysregulation in prurigo nodularis compared with psoriasis and atopic dermatitis. J Invest Dermatol.

[21] Vasavda C., Wan G., Szeto M.D. (2023). A polygenic risk score for predicting racial and genetic susceptibility to prurigo nodularis. J Invest Dermatol.

[22] Beck L.A., Thaçi D., Hamilton J.D. (2014). Dupilumab treatment in adults with moderate-to-severe atopic dermatitis. N Engl J Med.

[23] Blauvelt A., de Bruin-Weller M., Gooderham M. (2017). Long-term management of moderate-to-severe atopic dermatitis with dupilumab and concomitant topical corticosteroids (LIBERTY AD CHRONOS): a 1-year, randomised, double-blinded, placebo-controlled, phase 3 trial. Lancet.

[24] Bachert C., Han J.K., Desrosiers M. (2019). Efficacy and safety of dupilumab in patients with severe chronic rhinosinusitis with nasal polyps (LIBERTY NP SINUS-24 and LIBERTY NP SINUS-52): results from two multicentre, randomised, double-blind, placebo-controlled, parallel-group phase 3 trials. Lancet.

[25] Rabe K.F., Nair P., Brusselle G. (2018). Efficacy and safety of dupilumab in glucocorticoid-dependent severe asthma. N Engl J Med.

[26] Akinlade B., Guttman-Yassky E., de Bruin-Weller M. (2019). Conjunctivitis in dupilumab clinical trials. Br J Dermatol.

[27] Eichenfield L.F., Bieber T., Beck L.A. (2019). Infections in dupilumab clinical trials in atopic dermatitis: a comprehensive pooled analysis. Am J Clin Dermatol.

[28] Blauvelt A., Wollenberg A., Eichenfield L.F. (2023). No increased risk in overall infection in adults with moderate-to-severe atopic dermatitis treated for up to 4 years with dupilumab. Adv Ther.

[29] Paller A.S., Beck L.A., Blauvelt A. (2022). Infections in children and adolescents treated with dupilumab in pediatric clinical trials for atopic dermatitis – a pooled analysis of trial data. Pediatr Dermatol.

[30] Silverberg J.I., Yosipovitch G., Simpson E.L. (2020). Dupilumab treatment results in early and sustained improvements in itch in adolescents and adults with moderate to severe atopic dermatitis: analysis of the randomized phase 3 studies SOLO 1 and SOLO 2, AD ADOL, and CHRONOS. J Am Acad Dermatol.

[31] Berdyshev E., Goleva E., Bissonnette R. (2022). Dupilumab significantly improves skin barrier function in patients with moderate-to-severe atopic dermatitis. Allergy.

[32] Garcovich S., Maurelli M., Gisondi P. (2021). Pruritus as a distinctive feature of type 2 inflammation. Vaccines (Basel).

[33] Oetjen L.K., Mack M.R., Feng J. (2017). Sensory neurons co-opt classical immune signaling pathways to mediate chronic itch. Cell.

[34] Wang F., Kim B.S. (2020). Itch: a paradigm of neuroimmune crosstalk. Immunity.

[35] Mack M.R., Miron Y., Chen F. (2023). Type 2 cytokines sensitize human sensory neurons to itch-associated stimuli. Front Mol Neurosci.

